# An Institutional Celebrity's Birthday

**Published:** 1913-02-01

**Authors:** 


### An Institutional Celebrity's Birthday.

In recounting the actions or writing of the lives of individuals who in their lifetime have taken an active interest in the various institutions with which they are or were connected, there is one name that, in my opinion, writes a correspondent, stands over his compeers in this respect. The gentleman to whom I refer is Mr. H. L. Hargraves, J.P., who is closely connected with the Oldham Royal Infirmary. Mr. Hargraves will have attained the age of eighty-six on the 10th of February next, and although advanced in years his mental faculties remain unimpaired. He claims to be, if not the oldest, one of the oldest living members of the British Pharmaceutical Society, having become an associate in the year 1841, and a few years later a member. It is interesting to recall that after retiring from the business of a chemist and druggist (over twenty years ago) he became a governor of the hospital mentioned, and has during this period purchased the whole of the drugs, dressings, and so forth, which are used in the institution. It is his boast that he and his father have dealt continuously with what is now known as The British Drug Houses, Ltd., London, for over 100 years. He may be seen daily going round the wards of the institution cheering and comforting the patients both by kindly words and financial help to the needy. He has from time to time given to the infirmary over £7,000, in sums ranging from £50 to £5,000, besides gifts in kind, chief amongst the latter being a grand piano for each of the eight wards in the institution. In addition to this he gave during the past year a sum of £200, to be invested at 4 per cent., the interest of which is to be used in providing gold medals for the most successful nurses (theoretical and practical) in their respective years of probation, and also paid for the entire renovation of the mortuary. At the last meeting of the general committee, a few days ago, he proffered to purchase a new ambulance of the most modern type at a cost of £150 to replace the present worn-out one.

Mr. Hargraves's beneficence has not been confined to the Oldham Royal Infirmary, but the borough has in its Art Gallery many valuable pictures given by him, together with playgrounds and open spaces for the people, whilst his old-folks' parties have become a feature in Oldham.

